# Prognostic Function of Programmed Cell Death-Ligand 1 in Esophageal Squamous Cell Carcinoma Patients Without Preoperative Therapy: A Systematic Review and Meta-Analysis

**DOI:** 10.3389/fonc.2021.693886

**Published:** 2021-08-18

**Authors:** Hongxia Cui, Yarong Li, Su Li, Guangxuan Liu

**Affiliations:** ^1^Department of Pharmacy, Cancer Hospital of China Medical University, Liaoning Cancer Hospital and Institute, Shenyang, China; ^2^School of Life Science and Biopharmaceutics, Shenyang Pharmaceutical University, Shenyang, China

**Keywords:** esophageal squamous cell carcinoma (ESCC), programmed cell death-ligand 1 (PD-L1), clinicopathological features, overall survival, meta-analysis

## Abstract

**Background:**

Studies investigating the correlation between the expression of programmed cell death-ligand 1 (PD-L1) and prognosis in patients with esophageal squamous cell carcinoma (ESCC) not receiving preoperative therapy have increased significantly, but conclusions remain inconclusive. Therefore, this study aimed to determine the association between clinical outcomes and expression of PD-L1 in ESCC patients without preoperative therapy.

**Methods:**

We conducted a comprehensive literature search using four databases up to May 2020. Quality assessment was carried out according to the Newcastle–Ottawa Quality Assessment Scale (NOS). Hazard ratios (HRs) were used to analyze the association between PD-L1 expression with prognosis. Furthermore, we evaluated the correlation between PD-L1 and clinicopathological characteristics using odds ratios (ORs) and 95% confidence intervals (CIs).

**Results:**

Twenty studies (19 publications) comprising 3,677 patients were included in this meta-analysis. We found that the expression of PD-L1 was not related to overall survival (OS, HR: 1.16, 95% CI: 0.94–1.42, *p* = 0.16) or disease-free survival (DFS, HR: 0.85, 95% CI: 0.66–1.10, *p* = 0.21) in ESCC. Furthermore, although PD-L1 expression was not significantly associated with sex, degree of differentiation, TNM stage, T stage, lymph node status, smoking, or alcohol use, the merged OR demonstrated that the expression of PD-L1 was higher in older patients compared to younger patients (OR: 1.40, 95% CI: 1.07–1.83, *p* = 0.01). No obvious publication bias was observed.

**Conclusions:**

Our present study illustrated that PD-L1 expression was not related to poor prognosis of ESCC patients not receiving preoperative therapy, albeit the association only showed a tendency for statistical significance. Notably, PD−L1 expression showed a significant association with age. This meta-analysis had several limitations; therefore, our results need to be verified through further large-scale and prospective studies.

## Introduction

Esophageal cancer (EC) is ranked as the seventh most common cancer worldwide and was the sixth leading cause of death from cancer in 2018 (1). Esophageal squamous cell carcinoma (ESCC) and esophageal adenocarcinoma (EAC) are the two main histological types of EC, with ESCC accounting for approximately 90% of all EC (2). Recently, accumulated evidence has shown that EAC and ESCC are two different diseases, as the profiles of genomic alterations differ widely in the two diseases and they have completely different risk factors (3). There are marked geographic differences in the incidence of ESCC. The prevalence of ESCC is high in East Asia, East Africa, South Africa, and southern Europe. In contrast, ESCC has a relatively low incidence rate in North America and other parts of Europe (4). Currently, there are many different treatments for patients with ESCC, including endoscopic therapy, surgery, chemotherapy, and radiotherapy. Although the development of comprehensive treatment can improve survival, the 5-year survival rate of ESCC is only 15% (5). Currently, there is no gold standard to predict clinical survival or guide the choice of treatment strategy. Therefore, it is of interest to identify valuable prognostic markers for patients with ESCC.

Immunotherapy has changed the standard and concept of tumor treatment, and it has become the third revolution of tumor therapy after traditional chemotherapy drugs and targeted therapy (6). Immunotherapy based on immune checkpoint inhibitors has shown unprecedented safety and efficiency in the treatment of a variety of immunogenic tumors and durable responses can be achieved in non-small cell lung cancer and malignant melanoma (7, 8). Programmed cell death-ligand 1 (PD-L1), one of the immune checkpoint molecules, is also known as the B7 homolog 1 (B7-H1) or leukocyte differentiation antigen 274 (CD274). PD-L1 is a protein that is encoded by the CD274 gene. The combination of PD-L1 on tumor cells and its receptor PD-1 on T cells interferes with the activity of the PI3K/AKT and Ras/MEK/ERK signaling pathways, and as a result, T-cell proliferation, activation, and survival are impaired (9). Besides cancer cells, macrophages, dendritic cells, and cancer-associated fibroblasts can also express PD-L1. These components create an immunosuppressive microenvironment, which leads to tumor immune escape. PD-L1 can also promote the secretion of several cytokines, such as tumor necrosis factor (TNF)-α, IL-1β, IL-6, and IL-8 (10), and as a result, PD-L1 can promote immune response. Immunotherapy can restore anti-tumor immune response, which can control and clear tumor cells (11). Currently, multiple meta-analyses have shown that the expression of PD-L1 on tumor cells exhibits predictive value for prognosis in a variety of tumors, including gastric cancer (12), malignant pleural mesothelioma (13), bone and soft tissue sarcomas (14), colorectal cancer (15, 16), glioblastoma (17), nasopharyngeal carcinoma (18), and small cell lung cancer (19).

Recently, studies evaluating the association between PD-L1 with clinical outcomes and variables in ESCC patients have increased significantly. However, the overall conclusion remains uncertain. Some studies have shown that the expression of PD-L1 was correlated with an unfavorable survival in ESCC (20–27), while others have reported the opposite results (28, 29). Several meta-analyses have studied the prognostic role of PD-L1 in patients with ESCC. Qu et al. found a trend for PD-L1 association with shorter OS, but this was not statistically significant (30). However, Guo et al. suggested that PD-L1 was an indicator of shorter OS, and thus, a potential biomarker for prognosis. Notably, Guo et al. conducted subgroup analysis stratifying patients according to whether they had received preoperative neoadjuvant therapy. The subgroup analysis of the nine studies without neoadjuvant treatment illustrated that PD-L1 expression was not associated with OS, which was different from the conclusion taking into consideration the entire study population (31). Therefore, the purpose of our present study was to summarize the prognostic significance of PD-L1 expression by tumor cells in ESCC patients without preoperative therapy. Our results may help researchers to identify whether PD-L1 expression could be applied as a valuable prognostic predictor in ESCC.

## Materials and Methods

### Literature Search

This study was performed based on the Preferred Reporting Items for Systematic Reviews and Meta-Analysis guidelines (32). We conducted a comprehensive literature search using four databases, namely, PubMed, Cochrane Library, EMBASE, and Web of Science (from the establishment of the database to May 2020). The following search strings and Boolean operators were used: (“B7H1 Immune Costimulatory Protein” or “CD274 Antigens” or “CD274 Antigen” or “Programmed Cell Death 1 Ligand 1 Protein” or “PD L1 Costimulatory Protein” or “PD-L1 Costimulatory Protein” or “B7 H1 Immune Costimulatory Protein” or “B7-H1 Immune Costimulatory Protein” or “Programmed Cell Death 1 Ligand 1” or “B7 H1 Antigen” or “B7-H1 Antigen”) and (“Esophageal Neoplasms” or “Esophageal Cancers” or “Esophageal Cancer” or “Esophagus Cancers” or “Esophagus Cancer” or “Cancer of the Esophagus” or “Cancer of Esophagus” or “Esophagus Neoplasms” or “Esophagus Neoplasm” or “Esophageal Neoplasm”). In order to screen additional eligible studies, we also manually searched the references in the available studies. This review was registered in the PROSPERO database (CRD42020176354).

### Study Selection

Qualified studies were carefully selected in accordance with the following inclusion criteria: (1) patients were confirmed ESCC by pathological diagnosis; (2) the detection method of PD-L1 expression was immunohistochemistry (IHC); (3) PD-L1 expressed by tumor cells was detected; (4) studies reported directly hazard ratios (HRs) and 95% confidence intervals (95% CIs) for overall survival (OS) or disease-free survival (DFS), or sufficient data were provided to calculate the HR and 95% CIs; and (5) studies provided data to calculate odds ratio (OR) and 95% CIs for the correlation of PD-L1 expression with clinical parameters. Articles that met any of the following criteria were excluded: (1) studies including patients who received preoperative treatment; (2) duplicate publications or non-English articles; (3) conference abstracts, case reports, review articles, comments, or letters; or (4) experiments not in humans.

### Data Extraction

Two authors independently extracted information from eligible articles. The authors resolved differences through discussion. The extracted data were as follows: (1) basic characteristics (first author, country, year of publication, tumor stage, sample size, rate of PD-L1 expression, antibodies for PD-L1 detection, cutoff value, and detection method); (2) HRs and 95% CIs were extracted for OS or DFS. If the studies did not directly report HRs and 95% CIs, we calculated the data based on the methods described by a previous article (33); (3) the number of patients for each clinicopathological feature was extracted. If the relevant information of some items was not provided, these items were marked as “not available” (“NA”). When the HRs for the survival outcomes were reported by both univariate and multivariate analysis, only the HRs from the multivariate analysis were extracted.

### Quality Assessment of Included Studies

Quality assessment of the retrieved articles was carried out in accordance with the Newcastle–Ottawa Quality Assessment Scale (NOS) by two independent reviewers. The evaluation categories mainly included selection, comparability, and outcome (cohort study). The scores for each category were 0–4, 0–2, and 0–3. The maximum score was 9. The articles were considered to be eligible if their scores were ≥6.

### Statistical Analysis

Pooled HRs were adopted to investigate the correlation between the PD-L1 expression and survival outcomes of ESCC. The relationship between PD-L1 expression with clinical features was revealed by ORs. The *I* ^2^ and Q test were applied to quantify the heterogeneity between eligible studies. If there was no significant heterogeneity (*I* ^2^ < 50% or *p* > 0.10), we chose a fixed effects model. Otherwise, we adopted a random effects model. Additionally, we conducted a subgroup meta-analysis to discuss the underlying heterogeneity. The Begg’s test was employed to analyze potential publication bias. We used STATA version 14.0 software and Review Manager Version 5.3 to analyze the data.

## Results

### Selection of Studies

**Figure 1:**
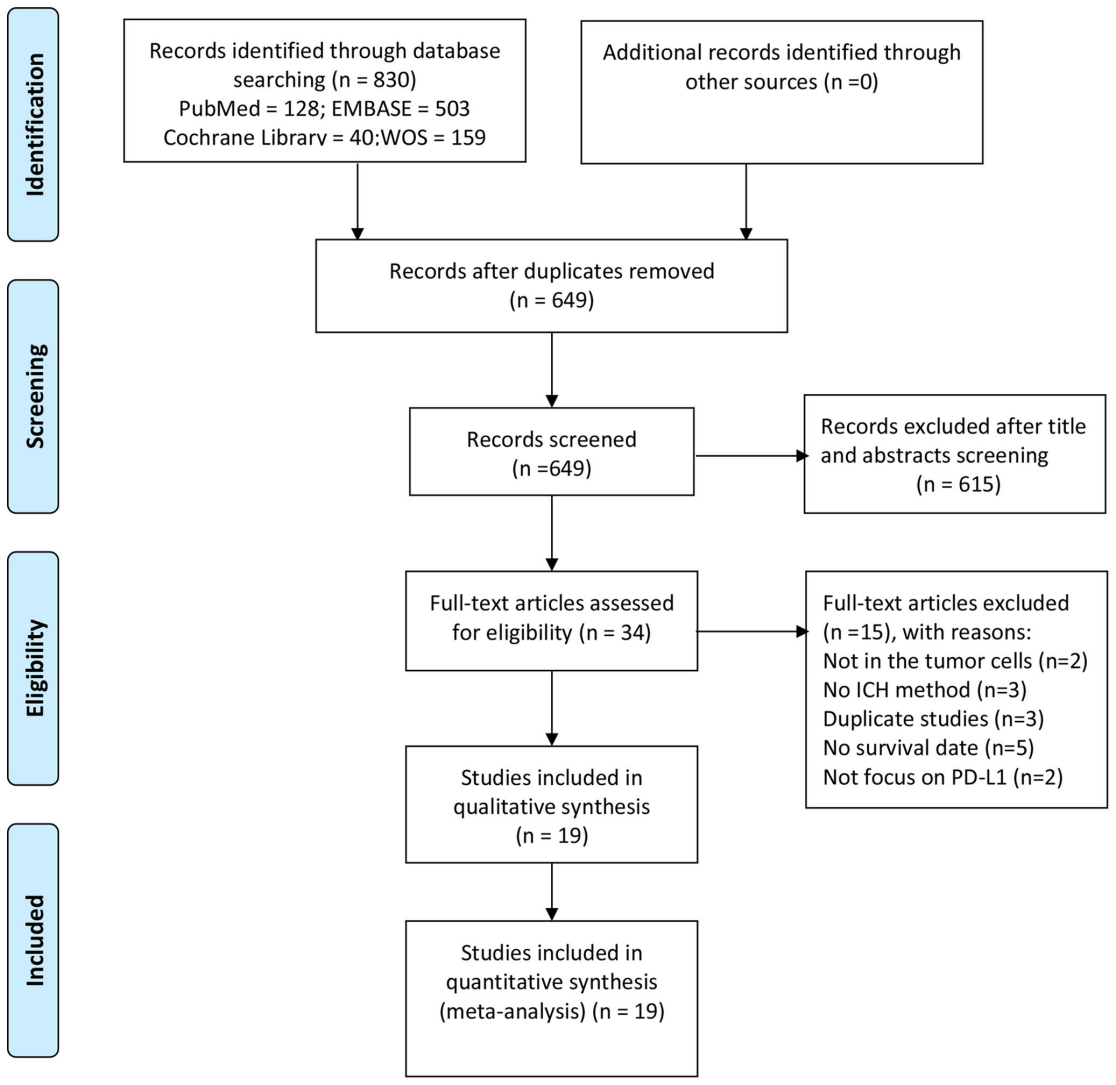
Procedure of literature screening.

In total, 830 studies were retrieved from the literature based on the abovementioned search strategy. After the exclusion of duplicate studies, 649 studies were left. Next, we excluded 615 studies by reviewing titles and abstracts. Subsequently, the remaining 34 studies were screened for further full-text examination. Of these, 15 studies were removed because of the selection criteria (in two studies, PD-L1 was not expressed by tumor cells, three articles did not employ IHC to detect PD-L1, three articles used the same sample population, five articles did not provide available information, and two articles did not focus on PD-L1). Finally, 19 publications (20–29, 34–42) containing 20 independent studies comprising a total of 3,677 patients were selected for our study. The process of study selection is summarized in **Figure 1**.

### Characteristics of the Studies

**Table 1** presents the details of each eligible study. Briefly, the included studies were published between 2014 and 2020. Thirteen reports were carried out in China (20, 21, 23–28, 34, 37–39, 42), five in Japan (22, 35, 40, 41), one in Taiwan (36), and one in Germany (29). The median number of patients was 184 for all eligible studies (range, 72 to 536). IHC was adopted to detect the expression of PD-L1 in all studies, but the IHC protocols used different between the included studies in terms of different antibodies and cutoff values. All 20 studies provided data on OS (11 directly reported HRs by multivariate analysis, 2 directly provided HRs by univariate analysis, and 7 only presented survival curves). In terms of the DFS, 10 studies reported data on DFS (five reported HRs by multivariate analysis, two directly reported HRs by univariate analysis, and three only provided survival curves). Of all the included studies, the scores of quality assessment were ≥6 according to NOS (eight studies scored 8, six scored 7, and six studies scored 6), which indicated that all enrolled articles were of high quality.

**Table 1:** Main characteristics of eligible literature.

Author	Year	Country	Stage	No. of pts	Age (years)	Positive PD-L1 (%)	Antibody				Cutoff value	Assay method	Outcome	NOS score
							Company	Source	Type	Clone				
Chen K (34)	2016	China	I–IV	536	60 (37–77)	41.4 (222/536)	Sigma-Aldrich, Saint Louis, USA	Rabbit	NA	SAB2900365	≥5%	IHC	OS/DFS	8
Chen L (20)	2014	China	I–IV	99	59	82.8 (82/99)	Novus Biologicals Littleton, CO, USA	Rabbit	MAB	NBP1-03220	H-score > 0	IHC	OS	6
Duan (21)	2018	China	I–IV	95	58 (38–81)	31.6 (30/95)	Signaling Technology	Rabbit	MAB	E1L3N	≥10%	IHC	OS	7
Guo (28)	2018	China	I–III	233	60 (36–85)	55.4 (129/233)	Abcam, Cambridge, UK	Rabbit	MAB	28-8	IRS (0-9) ≥3	IHC	OS/DFS	7
Hatogai (35)	2020	Japan	I–IV	192	66 (42–87)	22.4 (43/192)	NA	Rabbit	MAB	E1L3N	≥1%	IHC	OS	6
Hsieh (36)	2018	Taipei	I–IV	150	64.1 ± 10.8 (36–88)	64.0 (96/150)	BioLegend	Mouse	MAB	329702	Staining intensity 0, 1+, 2+, 3+, cutoff value of ≥score 2	IHC	OS/DFS	7
ITo S (22)	2016	Japan	NA	90	62.7 (38–82)	18.9 (17/90)	Lifespan Biosciences, Seattle, WA	Rabbit	PAB	Cat. no. LS-B480	scores=added of area and intensity (0–8). Cut off ≥7	IHC	OS	7
Jesinghaus (29)	2017	Germany	I–IV	125	60 (39–83)	30.4 (38/125)	VENTANA	Rabbit	MAB	SP263	>10%	IHC	OS/DFS	8
Jiang D (38)	2017	China	I–IVa	278	62 (37–83)	50.7 (141/278)	OriGene Technologies, Maryland, USA	Rabbit	MAB	SP142	≥1%	IHC	OS/DFS	8
Jiang C (37)	2019	China	II–III	246	58 (37–80)	24.4 (60/246)	Cell Signaling Technology, Inc., Danvers, MA, USA	Rabbit	MAB	E1L3N	H-score>15	IHC	OS	8
Leng (23)	2016	China	I–IV	106	59 (38–80)	53.8 (57/106)	Abcam, Cambridge, MA, USA	Rabbit	PAB	ab58810	IRS (0-9) >3	IHC	OS	6
Liang (24)	2020	China	I–IV	105	NA	44.8 (47/105)	Cambridge, UK	Rabbit	MAB	ab21-3524	IRS (0-12) > 4	IHC	OS	7
Rong (39)	2019	China	NA	378	NA	29.9 (113/378)	Spring Bioscience, Pleasanton, CA, USA	Rabbit	MAB	SP142	≥1%	IHC	OS/DFS	8
Tsutsumi (40)	2017	Japan	NA	90	62.7	63.3 (57/90)	Lifespan Bioscience, Seattle, WA, USA	Rabbit	PAB	NA	≥5%	IHC	OS	6
Wakita (41)	2017	Japan	IB–IIIC	72	NA	15.7 (15/72)	Cell Signaling Technology, Danvers, MA	Rabbit	MAB	13684	≥10%	IHC	OS	8
Wakita (41)	2017	Japan	IB–IIIC	105	NA	32.4 (34/105)	Cell Signaling Technology, Danvers, MA	Rabbit	MAB	13685	≥10%	IHC	OS	8
Wang (25)	2018	China	I–III	146	59.1 (37–78)	61.7 (90/146)	Cell Signaling Technology, USA	Rabbit	MAB	ab13684S	IRS (0-9) ≥ 3	IHC	OS	6
Zhang (42)	2017	China	II–III	344	NA	14.5 (50/344)	Spring Bioscience, Pleasanton, CA, USA	Rabbit	MAB	SP142	≥5%	IHC	OS/DFS	8
Zhao (26)	2018	China	I–IV	154	55 (37–48)	53.9 (83/154)	Abcam	NA	NA	NA	≥10%	IHC	OS	7
Zhu (27)	2016	China	II	133	59	41.3 (56/133)	Beijing Zhongshan Golden Bridge Company	Rabbit	MAB	SP142	Membranous or cytoplasmic staining was observed in TCs	IHC	OS/DFS	6

NA, not available; PAB, polyclonal antibody; MAB, monoclonal antibody; IHC, immunohistochemistry staining; OS, overall survival; DFS, disease-free survival.

### Correlation Between PD-L1 Expression and OS

**Figure 2:**
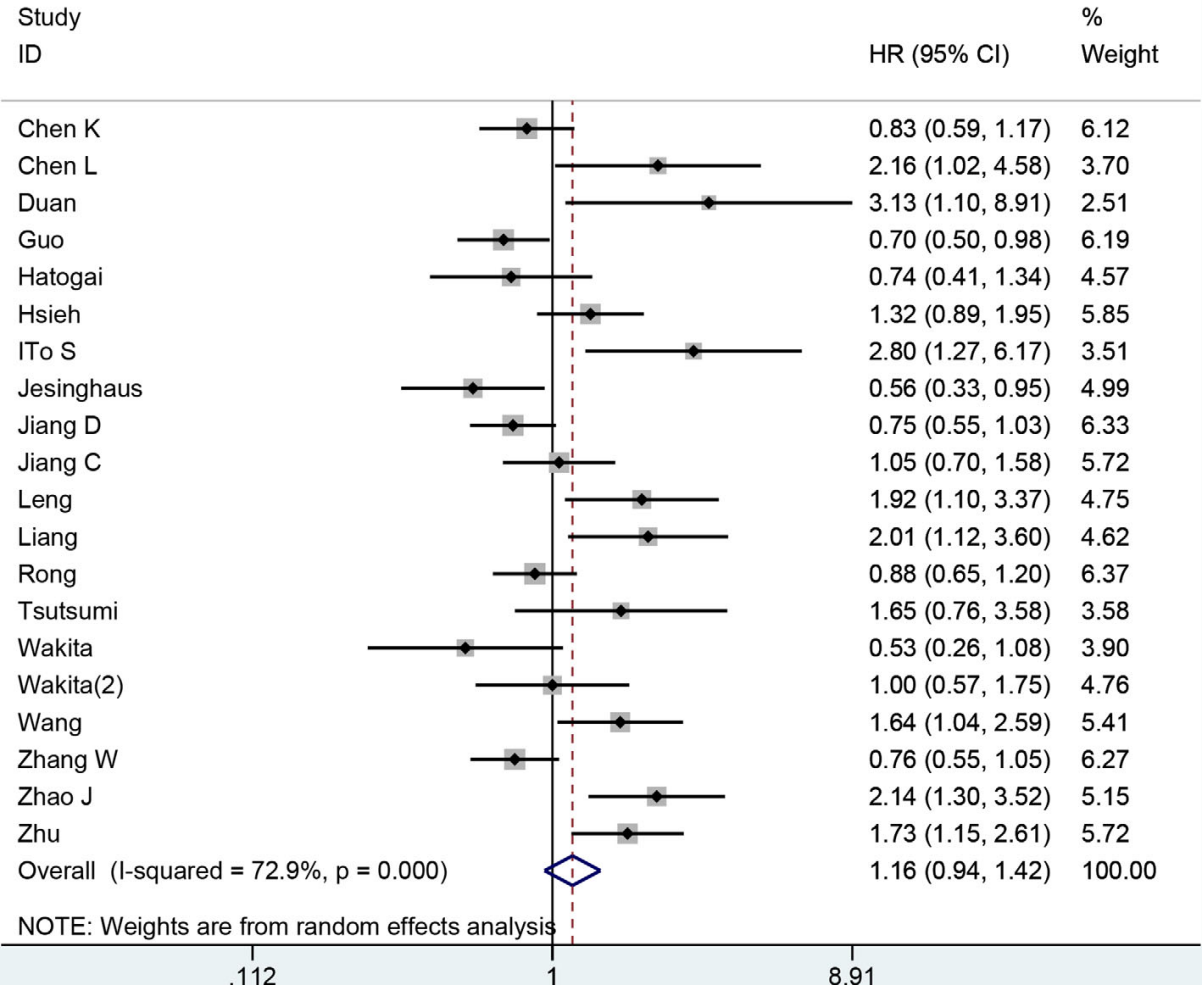
Forest plot for the association of PD-L1 with overall survival in ESCC.

We pooled the relevant data from 20 studies (19 publications) comprising 3,677 patients to investigate the association between PD-L1 expression and OS. We employed a random effects model to analyze data for significant heterogeneity (*I* ^2^ = 72%, *p* < 0.001). No significant relationship was found between the PD-L1 expression and OS in ESCC patients (HR: 1.16, 95% CI: 0.94–1.42, *p* = 0.16) (**Figure 2**). To determine the source of heterogeneity among included studies, subgroup analyses were conducted by calculating HRs, sample size, and region. The subgroup analyses showed that these factors were not the causes of heterogeneity (**Table 2**). The subgroup analysis based on sample size revealed that higher expression of PD-L1 was associated with shorter OS when the sample size was ≤160 (HR: 1.49, 95% CI: 1.14–1.95, *p* = 0.003). However, we observed longer OS in patients with higher PD-L1 expression when the sample size was >160 (HR: 0.81, 95% CI: 0.71–0.92, *p* = 0.009). When we performed subgroup analyses stratified by calculation of HRs and study region, high PD-L1 expression was not associated with OS.

**Table 2:** Subgroup analysis of the correlation between PD-L1 expression and overall survival in ESCC.

Subgroup	Number of	Statistical	HR (95%CI)	*p*-value	Heterogeneity	
	studies	model			*I*^2^ (%)	*p*-value
OS	20	Random	1.16 (0.94–1.42)	0.160	73	<0.001
**Calculation of HRs**						
Multivariate	11	Random	1.22 (0.92–1.61)	0.170	76	<0.001
Univariate	9	Random	1.09 (0.80–1.49)	0.58	71	<0.001
**Sample Size**						
≤160	13	Random	1.49 (1.14–1.95)	0.003	64	<0.001
>160	7	Fixed	0.81 (0.71–0.92)	0.009	0	0.430
**Region**						
China	14	Random	1.23 (0.98–1.55)	0.070	75	<0.001
Japan	5	Random	1.09 (0.64–1.85)	0.750	67	0.020
Germany	1	–	0.56 (0.33–0.95)	0.030	–	–
DFS	10	Random	0.85 (0.66–1.10)	0.210	73	<0.001
**Calculation of HRs**						
Multivariate	5	Random	0.85 (0.58–1.24)	0.390	81	<0.001
Univariate	5	Random	0.85 (0.59–1.24)	0.410	68	0.010
**Sample Size**						
≤160	5	Random	1.05 (0.83–1.33)	0.680	85	<0.001
>160	5	Fixed	0.82 (0.71–0.95)	0.009	0	0.430
**Region**						
China	7	Random	0.97 (0.76–1.23)	0.78	69	<0.001
Japan	2	Random	0.60 (0.20–1.78)	0.36	76	0.040
Germany	1	–	0.38 (0.21–0.68)	0.001	–	–

HR, hazard ratio.

### Correlation of PD-L1 Expression With DFS

**Figure 3:**
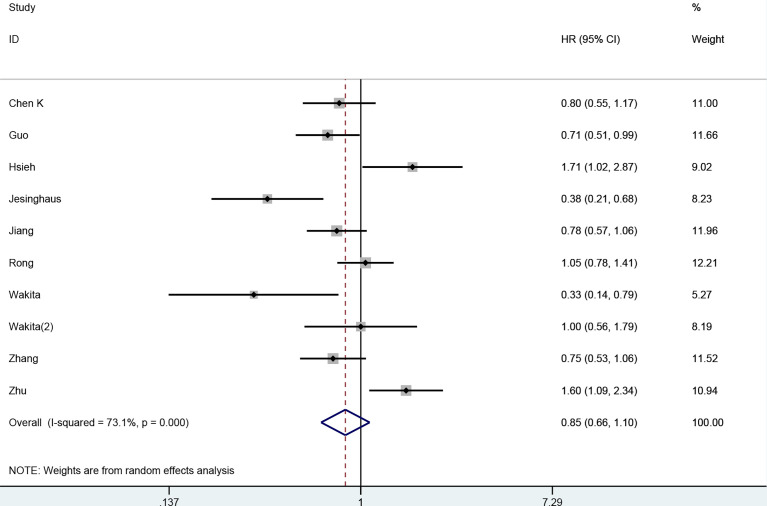
Forest plot for the association of PD-L1 with disease-free survival in ESCC.

In total, 10 studies (nine publications) comprising 2,354 patients reported the correlation of PD-L1 expression with DFS. A random effects model was employed, as we observed obvious heterogeneity (*I* ^2^ = 73%, *p* < 0.001). Significant predictive effects of PD-L1 expression on the DFS were not observed in ESCC (HR: 0.85, 95% CI: 0.66 to 1.10, *p* = 0.21) (**Figure 3**). The subgroup analyses by calculation of HRs, sample size, and study region did not reduce heterogeneity (**Table 2**). The result of subgroup analysis by sample size revealed that the positive expression PD-L1 was correlated with better DFS when sample size was >160 (HR: 0.82, 95% CI: 0.71–0.95, *p* = 0.009). However, subgroup analyses stratified by calculation of HRs and study region suggested that PD-L1 expression was not related to DFS.

### Clinicopathological Features

The association of PD-L1 expression with clinicopathological characteristics is summarized in **Table 3**. We mainly evaluated the following characteristics: age, sex, tumor differentiation, TNM stage, T stage, lymph node status, smoking, and alcohol use. The merged OR revealed that PD-L1 expression in older ESCC patients was higher than that in younger ESCC patients (OR: 1.40, 95% CI: 1.07–1.83, *p* = 0.01). However, expression of PD-L1 had no significant correlation with sex (OR: 1.03, 95% CI: 0.84–1.26, *p* = 0.76), differentiation (OR: 1.18, 95% CI: 0.80–1.75, *p* = 0.41), TNM stage (OR: 1.11, 95% CI: 0.76–51.61, *p* = 0.59), T stage (OR: 1.30, 95% CI: 0.74–2.25, *p* = 0.36), lymph node status (OR: 1.32, 95% CI: 0.86–2.02, *p* = 0.21), smoking history (OR: 1.06, 95% CI: 0.81–1.38, *p* = 0.68), or alcohol use (OR: 0.98, 95% CI: 0.70–1.36, *p* = 0.89).

**Table 3:** The correlations of PD-L1 expression with clinicopathological features of ESCC.

Characteristics	Number of studies	Statistical model	OR (95% CI)	*p*-value	Heterogeneity	
					*I*^2^ (%)	*p*-value
Sex (male *vs.* female)	16	Fixed	1.03 (0.84–1.26)	0.760	2	0.430
Age (years) (≥60 *vs.*<60)	8	Fixed	1.40 (1.07–1.83)	0.010	0	0.530
Differentiation (poor *vs.* moderate/well)	13	Random	1.18 (0.80–1.75)	0.410	65	<0.001
TNM stage (III–IV *vs.* I–II)	12	Random	1.11 (0.76–1.61)	0.590	72	<0.001
T stage (T3–T4 *vs.* T1–T2)	12	Random	1.30 (0.74–2.25)	0.360	85	<0.001
Lymph node status (positive *vs.* negative)	13	Random	1.32 (0.86–2.02)	0.210	81	<0.001
Smoking (yes *vs.* no)	4	Fixed	1.06 (0.81–1.38)	0.680	0	0.810
Alcohol use (yes *vs.* no)	2	Fixed	0.98 (0.70–1.36)	0.890	0	0.820

### Publication Bias

**Figure 4:**
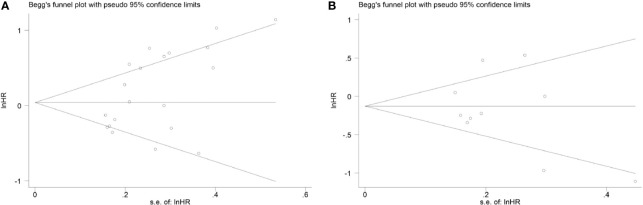
Begg’s funnel plot of publication bias **(A)** for overall survival and **(B)** for disease-free survival.

The Begg’s test was employed to assess whether there was publication bias. The results confirmed that there was no significant publication bias for OS (*p* = 0.064, **Figure 4A**) or DFS (*p* = 0.721, **Figure 4B**), which supported the robustness of our results.

### Sensitivity Analysis

**Figure 5:**
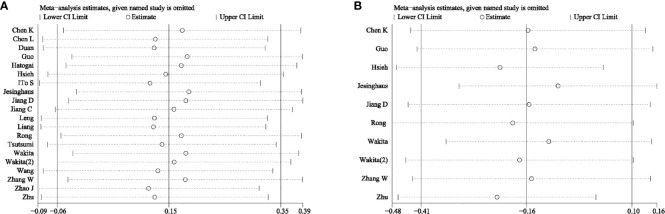
Sensitivity analysis **(A)** for overall survival and **(B)** for disease-free survival.

To further examine the robustness of the prognostic potential of PD-L1, we carried out sensitivity analysis. There was no individual study that could significantly influence the pooled HRs, which suggested that the results were credible (**Figure 5**).

### Meta Regression Analysis

Meta regression analysis was carried out to investigate the potential source of heterogeneity. Meta regression analysis for OS indicated that the sample size (*p* = 0.027) may have contributed to heterogeneity. However, the calculation of HRs (*p* = 0.537) and study region (*p* = 0.146) did not affect heterogeneity. Meta regression analysis for DFS showed that the sample size (*p* = 0.548), calculation of HRs (*p* = 0.425), and study region (*p* = 0.570) were not the sources of the heterogeneity of DFS.

## Discussion

Treatment with immune checkpoint inhibitors could be used to rescue the suppressed tumor-killing immune response (43). As a form of immunotherapy, PD-1/PD-L1 checkpoint inhibitors have shown good efficacy in ESCC patients. The KEYNOTE-180 clinical trial showed that the objective response rates (ORR) of ESCC and EAC patients were respectively 14.3% and 5.2% after pembrolizumab monotherapy. In addition, the ORR for PD-L1-positive patients and for PD-L1-negative patients was 13.8% and 6.3%, respectively, which suggested that pembrolizumab was effective for ESCC patients with PD-L1 (44). Therefore, pembrolizumab has been approved as a second-line treatment for advanced ESCC patients with PD-L1.

The phase III trial ATTRACTION-03 showed that compared with chemotherapy alone, nivolumab prolonged OS in advanced ESCC patients (10.9 months *vs*. 8.4 months). Moreover, patients with advanced ESCC could also benefit from treatment with nivolumab, regardless of the expression level of PD-L1 by tumor cells (45). This finding indicated that the PD-L1 expression might not be relevant to the prognosis of ESCC patients. Feng et al. determined that high expression of PD-L1 increased the risk of developing hepatocellular carcinoma in patients with cirrhosis, and patients with higher PD-L1 expression may have a worse prognosis than those with lower PD-L1 expression (46). Zheng et al. found that PD-L1 expression was related to lymph node metastasis and the degree of tumor differentiation in advanced gastric cancer patients (47). In brief, the PD-L1 expression was significantly associated with clinicopathological features and clinical outcomes; thus, PD-L1 expression could be used as an indicator of prognosis in a variety of tumors.

Nonetheless, conclusion regarding the prognostic potential of PD-L1 expression in ESCC patients without preoperative therapy is conflicting. Thus, we conducted this meta-analysis to provide evidence supporting a more definitive conclusion. We conducted a comprehensive literature search and included 19 publications containing 20 independent studies. We pooled the data to assess the effects of PD-L1 expression on OS, DFS, and clinicopathological features in ESCC patients. Overall, we demonstrated that PD-L1 expression showed no significant association with OS or DFS in patients with ESCC. Notably, for the 20 included studies, 10 studies suggested that PD-L1 expression had significant correlation with OS, while the other 10 studies reported that PD-L1 did not have any relationship with OS. However, The HR values of the 20 articles showed a similar contradictory tendency, which resulted in the negative results of the prognosis analysis.

PD-L1 plays different roles in the tumor microenvironment. PD-L1 expressed by tumor cells can inhibit the proliferation of T cells by transmitting negative regulatory signals and thus inhibit T-cell responses, which leads to tumor escape and promotes tumor growth (48). In contrast, PD-L1 can also induce T-cell proliferation and secretion of some cytokines such as IFN-γ and IL-10 by binding to unknown receptors, and as a result, PD-L1 can promote the immune response and anti-tumor (34, 41). We speculated that the above two effects of PD-L1 were in equilibrium in ESCC, leading to the absence of correlation between PD-L1 and prognosis. Furthermore, among the eight clinicopathological features evaluated, high PD-L1 expression correlated only with age, but not with sex, TNM stage, T stage, differentiation, lymph node metastatic status, smoking, or alcohol use, which indicated that the PD-L1 was a feature of the disease in older patients. We did not find publication bias using the Begg’s test and our results were reliable. As far as we know, this study was the first meta-analysis to explore the association between PD-L1 expression with prognosis and pathological features of ESCC patients not receiving preoperative treatment. The results of this study can provide an important basis for the selection of clinical treatment strategies for ESCC patients.

The combined results showed that although there was a trend for correlation between PD-L1 expression and prognosis, this difference was not statistically significant. However, half of the included studies reported that PD-L1 expression correlated with clinical outcomes. We found that the heterogeneity significantly reduced when we carried out subgroup analysis based on sample size, indicating that the sample size may be one of the reasons for heterogeneity. There may be several possible reasons for the high degree of heterogeneity in the present meta-analysis. Firstly, the cutoff values used to define PD-L1 expression, the antibodies used detection, and the criteria for immunohistochemical protocols were not consistent across the included studies, which may have represented the main source of heterogeneity. The percentage of positive cells, staining intensity, and combination of percentage of positive cells and staining intensity were employed to assess immunohistochemical results in the 20 articles. Future studies should standardize the cutoff values defining positive PD-L1 expression, detection antibodies used, and IHC staining protocols, to allow a better comparison of results obtained from different studies.

Secondly, another possible source of heterogeneity was the clinical stage. According to previous studies, the prognostic value of biomarkers differs depending on the clinical stage of the disease (49, 50). The clinical stages of the patients evaluated in the 20 included studies in our meta-analysis were not homogenous: 1 study enrolled patients with clinical stage II, included patients were limited to stages I to III in 4 studies and were limited to stages II to III in 2 studies, 10 studies included patients with clinical stages I to IV, while the remaining 3 studies did not provide any information on clinical stage. Therefore, it was difficult to conduct subgroup analysis by TNM stage. This inconsistency in reporting clinical stages needs to be evaluated in further prospective studies.

Thirdly, although we excluded patients who received preoperative therapy, few of the included studies mentioned whether the patients had received postoperative treatment, including adjuvant chemotherapy (AC) and immunotherapy. Only six studies mentioned that patients had received adjuvant treatment, and while the detailed treatment plans were not available, treatment regimens for the patients may contribute to heterogeneity. Different adjuvant treatment strategies post-surgery may also influence the survival of patients with ESCC and the tumor immune system, which may have influenced the analysis. Detailed non-surgical treatment information is necessary to assess the predictive effects of PD-L1 on survival outcomes; thus, we recommend that the treatment strategies need to be clearly identified in future prospective and retrospective studies.

Fourthly, host genetic and tumor genetic profiles may affect tumor staging, response to chemotherapy, and prognosis (51). Guo et al. (52) and Avincsal et al. (43) have revealed that the alcohol dehydrogenase 1B (ADH1B) gene polymorphism was associated with the prognosis of gastric cancer and hypopharyngeal cancer. Some studies have shown that PD-L1 gene polymorphisms are also associated with clinicopathological features and survival outcomes in a variety of tumors, such as lung cancer (53) and gastric cancer (54). In order to reduce the heterogeneity of the study findings, patients with similar genetic background should be included. Unfortunately, we could derive the genetic profile status of the included patients. Genetic heterogeneity of patients should be considered in future studies.

The prognostic potential of PD-L1 expression in patients with ESCC was also investigated in previous studies. A meta-analysis of eight articles involving 1350 ESCC patients established that positive PD-L1 expression could predict a shorter OS, although this was not shown to be statistically significant. Furthermore, expression of PD-L1 showed no correlation with sex, TNM stage, tumor depth, lymph node metastasis status, distal metastasis, or differentiation (30). Another meta-analysis including 13 articles indicated that high PD-L1 expression could predict a poor OS. Furthermore, high PD-L1 expression was related to distant metastasis in ESCC. However, expression of PD-L1 was not significantly correlated with DFS (31). Our meta-analysis revealed that higher expression of PD-L1 showed no correlation with OS or DFS in ESCC patients that had not received preoperative therapy and the expression of PD-L1 was higher in older ESCC patients. The findings of our meta-analysis were inconsistent with those of previous studies. These contrasting results were due to two main differences between previous meta-analysis and our study. Firstly, some recent articles have demonstrated that the prognostic potential of PD-L1 was associated to the neoadjuvant therapy strategy used, which included chemotherapy or radiotherapy (55–57). The previous two meta-analyses included only patients that had undergone preoperative therapy, whereas our meta-analysis excluded patients who had received preoperative therapy. This can eliminate the potential impact of preoperative therapy on the PD-L1 expression. Secondly, we included new studies published in 2019 and 2020 in our analysis. These new studies used different antibodies and different cutoff values for positive PD−L1 staining, which may have contributed to the different conclusions.

Although our study was conducted based on the PRISMA guidelines, there were several limitations that need to be noted. First, in some studies, we obtained HRs from Kaplan–Meier curves, which may have led to statistical bias. Second, the heterogeneity of our study was significant and could not be eliminated. Study design, PD-L1 antibodies, PD-L1 positivity cutoff values, and treatment strategies may have contributed to the heterogeneity. Third, although we did not establish inclusion criteria for the study design (retrospective or prospective) in the meta-analysis, the included studies were all retrospective in nature on literature screening. The predictive potential of PD-L1 expression warrants evaluation in future prospective studies.

## Conclusions

The findings of the present meta-analysis suggested that PD-L1 expression was not associated with poor prognosis of ESCC patients without preoperative therapy. Although this result was not significant, it showed a strong tendency towards statistical significance. Nevertheless, a significant correlation of PD-L1 expression with age was observed, with the expression of PD-L1 being higher in older ESCC patients. Given the several limitations of this meta-analysis, the prognostic potential of PD-L1 expression in ESCC patients without preoperative therapy remains to be definitively established and warrants verification through large-scale and prospective studies in the future.

## Data Availability Statement

The original contributions presented in the study are included in the article/supplementary material. Further inquiries can be directed to the corresponding author.

## Author Contributions

HC, YL, and SL conceived and designed the study. HC and YL carried out the literature search and data extraction. HC and YL analyzed the data and resolved the disagreement by discussion. HC, SL, and GL examined and verified the results. HC and YL wrote the paper. GL revised the whole manuscript. All authors contributed to the article and approved the submitted version.

## Conflict of Interest

The authors declare that the research was conducted in the absence of any commercial or financial relationships that could be construed as a potential conflict of interest.

## Publisher’s Note

All claims expressed in this article are solely those of the authors and do not necessarily represent those of their affiliated organizations, or those of the publisher, the editors and the reviewers. Any product that may be evaluated in this article, or claim that may be made by its manufacturer, is not guaranteed or endorsed by the publisher.
